# On a Case of Extra-Uterine Pregnancy: With Clinical Remarks

**Published:** 1867-08

**Authors:** Robert Greenhalgh

**Affiliations:** Physician-Accoucheur to St. Bartholomew’s Hospital, and Lecturer on Diseases of Women and Children; Consulting Physician to the City of London Lying-in Hospital, etc.


### SELECTIONS.

---

*On a Case of Extra-Uterine Pregnancy: with Clinical Remarks,—Favorable Termination.* By Robert Greenhalgh, M. D., Physician-Accoucheur to St. Bartholomew's Hospital, and Lecturer on diseases of Women and Children; Consulting Physician to the City of London Lying-in Hospital, etc.

The infrequency of extra-uterine pregnancy, the difficulties usually attending its diagnosis, the dangers present and prospective with which it is surrounded, and the insufficiency of all means hitherto devised for its arrest and safe termination will, I trust, be deemed sufficient grounds for occupying your time with the details of the following case, and with some reflections having a practical bearing upon this important class of cases.

On the 4th of last October, I was hurriedly summoned to the bedside of a lady who, I was informed, was suffering from the effects of extra-uterine pregnancy. I learnt, on my arrival at the house, that a general practitioner in the neighborhood had been hastily applied to on account of the patient having been seized with sudden acute pain, like cramp, in the lower abdomen. After a careful investigation, he somewhat indiscreetly informed the patient of what he believed to be the nature of the case and the danger of her condition, which so alarmed her and her husband, that they at once requested his withdrawal, on the score that so grave a case required a special practitioner I found the lady very pallid and greatly agitated. She was twenty-six years of age, and had been married just six months; had passed four catamenial periods, never having once "missed" since the establishment of that function, which had always been perfectly normal. About two months and a half ago she began to be very sick in the morning, when, after violent retching, she brought nothing but mucus, occasionally mixed with a little bile, off her stomach; she was never without a feeling of nausea, which, however, did not materially interfere with her appetite. Two months ago her breasts became tender and began to swell, and she noticed the nipples enlarging, and around them the skin becoming gradually darker; some small "pimples" at the same time making their appearance, she thought more marked on the left breast. Three months ago she experienced pain in the left groin and labium, sometimes extending to the inner and upper part of the thigh, which she described as a "bursting" sensation, accompanied at times, especially on exertion, with considerable irritability of the bladder and rectum. These symptoms gradually increased up to the time of my visit, when she was in the greatest suffering, so much increased by the slightest movement that she dreaded to shift her position. Having ascertained these facts I examined her breasts, which manifested all the usual characteristics of the pregnant condition at the third month, so ably depicted by Montgomery. Being placed on the back I proceeded to examine the abdomen, the parietes of which were thin and flaccid, when she screamed with agony, especially when pressure was made over the left iliac fossa.

Finding it impossible to make an accurate investigation, she was subsequently placed under the influence of chloroform, the bladder and bowels having been previously evacuated, when the nates were brought close to the edge of the bed, the thighs being flexed upon the abdomen, and her head propped up with pillows. The abdomen was tympanatic, except in the left iliac fossa, a little above and below Poupart's ligament, where there was a sense of resistance and slight dullness on percussion; a bruit could be distinctly heard about that region. Per vaginum, the vagina was somewhat elongated, the rugæ ill-developed; the cervix uteri slightly raised, enlarged and spongy to the touch; the margin of the os, granular to the feel and yielding. The cervix was driven to the right of the pelvis, the left being occupied by a round, ill-defined, elastic swelling, merging into the surrounding textures, which, upon pressure being made by the left hand, could be brought more easily within reach of the finger. I fancied more than once that I could detect something like a feeble *ballottement*. The uterus was slightly movable. At this stage of the investigation I was convinced of the existence of pregnancy, but that there was no embryo in the uterus. I therefore ventured carefully to introduce a sound into that viscus, which passed easily three inches in the normal direction, clearly proving that the uterus was increased in size. Per rectum, I could trace out the enlargement lying anteriorly and to the left of the canal, the uterus being forced over to the right of the pelvis. I may here remark, that this investigation was conducted with the greatest gentleness, as I feared the cyst might rupture, even with the most tender handling. The patient, being still partially under the influence of chloroform, was placed in a comfortable position, and the strictest quiet of mind and body was enjoined. I now informed her husband of the nature of the case and its hazards, confirming in every particular the opinion of the intelligent practitioner who had preceded me, at the same time requesting a consultation, which he (the husband) declined, saying that he was perfectly satisfied with the two opinions he had already had, and was ready to acquiesce in any plan of treatment I thought fit to recommend. I ordered a grain of morphia with cocoa-nut butter to be introduced into the rectum, an antispasmodic draught with hydrocyanic acid and small doses of morphia, ice and a tablespoonful of essence of meat to be administered from time to time.

On the morning of the 7th of October, I was pleased to find that my patient had passed two tranquil nights, and was in no way suffering from the recent examination. She had vomited twice with but slight efforts, had passed water, and had a slight sanious discharge from the vagina, with a sensation of bearing down. Pulse 86, feeble; lips dry; complains of thirst; skin slightly above the normal temperature, but moist; abdomen still tender. She had taken freely of meat-juice. Having thought over the case in all its aspects —the prospect of the gradual development of the ovum, with its daily increasing vascular supply, and the more than probable ultimate rupture of the cyst, giving rise to fatal internal hæmorrhage,—I determined to propose the puncture of the cyst through the roof of the vagina, and thus draw off the liquor amnii by means of a hair trocar and canula. Having fully explained to her husband the whole facts of the case and the method I proposed for its treatment, I gave him distinctly to understand that, although I had frequently, and with the best results, introduced that instrument into pelvic tumors, still I had had no experience whatever of its use, nor was I aware that it ever had been proposed, in a case similar to the one under discussion. Moreover, I stated that in the event of a consultation being held to determine the advisability of such a proceeding, I was fully prepared to meet with the stoutest opposition. Notwithstanding, I strongly urged a consultation, with the view, as I stated, of sharing the responsibility with another, he, however, declined to accede to my request.

At ten o'clock on the morning of the 8th I found my patient more comfortable, with less pain and tenderness in the abdomen, but much distressed by retching every now and then; she was, however, anxious to submit to the operation I proposed, and about which her husband had given her every information. Having carefully placed her in the ordinary obstetric position, I guided with the index-finger a long hair trocar and canula to the most accessible and depending part of the swelling, while gradual and firm pressure was made, by means of a large sponge, upon the left iliac fossa, so as to bring the tumor close against the roof of the vagina and within easier reach. I now plunged the trocar with canula firmly against the tumor, into which they entered to the extent of an inch, when, upon withdrawing the former, about fifteen drachms liquor amnii, slightly tinged with blood, escaped through the canula. I may here remark that when flowing, pressure on the abdomen was gradually discontinued, with the view of preventing the ingress of air. For a week my patient continued to progess most satisfactorily; the sickness and nausea, tenderness and pain, had gradually subsided, the breasts were decreasing in size, she was regaining her appetite, and her nervous agitation had almost wholly ceased; in short, to use her own expression, "she was quite another woman." On examining the left iliac fossa, it was found nearly, if not quite, as resonant as the right; there was no longer any sense of resistance to the touch, and she could bear tolerably firm pressure without flinching. The uterus had somewhat descended in the pelvis, and had assumed a more central position; and in place of the semi-eltastic and round swelling, a hard, irregular, well-defined, and slightly movable body could be felt to the left of that viscus.

She continued to improve up to October 27, when she was seized with a rigor, followed by pains in the hypogastric region of a paroxysmal character, accompanied with vomiting, acceleration of the pulse, and some heat of skin. These symptoms continued for about four hours, when a sanious discharge from the vagina made its appearance. During a severe paroxysm of pain some coagula were expelled, together with what proved to be, on careful examination, a portion of decidua, shortly after which she experienced almost perfect immunity from pain. From this time the discharge gradually ceased, having lasted five days; in fact, it was a menstrual period.

On the 3d of November last, I again made a careful vaginal examination, when I found the uterus nearly in its normal position and approaching its natural size. I could scarcely discover any trace of the extra-uterine swelling.

Nov. 6th, 1866.—The patient is fast regaining her strength, and is not conscious of any discomfort in or about the pelvis.

March 5th., 1867.—She is now in perfect health, having menstruated four times somewhat more freely than usual, but without pain.

It may now be interesting and instructive to you, gentlemen, if I append some few particulars of great practical importance connected with this subject. Dr. Campbell, in his excellent and critical monograph on Extra-Uterine Gestation, remarks: "After the extinction of its (the fœtus's) life, the fœtus has remained a long series of years in the abdomen of the parent with little inconvenience to her." He, however, suggested no method by which the progress of development can be arrested and such a result affected. Dr. Churchill says, "the child dies soon after the rupture of the cyst in most cases." Dr. Murphy remarks, "the treatment of such cases is out of our reach." Dr. F. H. Ramsbotham states, "Our treatment must depend entirely on the symptoms, and must be directed to the relief of pain and assisting nature in her efforts to get rid of the offending mass;" and further, "It cannot be necessary to lay down any rule of treatment for those cases in which the cyst bursts and contents pass into the abdominal cavity, because such are almost always rapidly fatal." Cazeau remarks: "When left to itself, an extra-uterine pregnancy will generally terminate in a rupture of the cyst.... This rupture, which is usually spontaneous, alwaysgives rise to extremely grave phenomena." And under the head of treatment, he observes: "It is evident that no operation could be attempted in the earlier months of pregnancy, even if we should be fortunate enough to ascertain with certainty that the ovule was not developed in the uterus." Dr. Hodge says, "In some cases the ovum perishes early, and the excitement and irritation diminish, and the embryo may remain encysted in its own membranes, not only for days or months but for years." This author further states that "the second termination is the rupture of the sac, the death of the fœtus, and usually the death of the mother from hæmorrhage or inflammation." Again: "Of all terminations, where the embryo perishes early, and is encysted in its own membranes, is by far the most favorable; as the tumor is small, and very little irritation is produced." Further: "It has been suggested to adopt measures as early as possible to destroy the life of the fœtus in extra uterine pregnancies, and thus to escape the accumulative dangers from rupture, hæmorrhage, or inflammation consequent upon its continued development;" adding, "the morality of this practice need not be defended, for it may be assumed that the child must sooner or later perish." And he records that in 1857 M. Bachetti of Pisa said that he had actually succeeded in destroying the life of the fœtus by electro-puncture, by inserting two needles into the sac and directing through them an electro-magnetic current.

I need not occupy more of your time by a further array of authorities to prove the dangers of these cases and the unsatisfactory treatment hitherto had recourse to.

Experience has demonstrated, amongst others, two most important facts: firstly, that in those cases in which the embryo perishes early, little or no further danger or evils need be apprehended; and secondly, that where its development is thus arrested, especially when enclosed within its own membranes, no more favorable issue could be desired.

It will be generally admitted that where the liquor amnii is discharged, either spontaneously or artificially, at an early period of gestation, embryonic life is almost if not quite certain to be extinguished, and the growth of the involucra arrested, and thus all fear of over-distension and rupture of the sac will be avoided. It may also be fairly conceded that the puncture of cysts about the cavity of the pelvis by means of a hair trocar and canula is rarely or never attended with serious results, especially if care be taken not to exhaust the sac by pressure upon the abdomen, thus avoiding the ingress of air. But it may be asked,—Is the diagnosis of extra-uterine pregnancy always so certain that we dare venture to introduce a trocar into a growth the nature of which may be so questionable? Doubtless, the diagnosis of these cases is frequently most obscure—nay, impossible; still, assuming the enlargement to be due to one or more of those growth usually found within the pelvis—such as pelvic abscess, hæmatocele, cyst of the broad ligament, ovarian dropsy, fibro-cystic tumor, &c.,—so much light would thereby, in all probability, be thrown upon the nature of the case as to determine our diagnosis, prognosis, and rational treatment, and in some few cases even more—the puncture may be, and has been, the very means by which the disease has been relieved, and in some instances cured.

If, then, we consider the danger of these progressive cases of extra-uterine pregnancy on the one hand, and the harmlessness of the means proposed and adopted for their arrest on the other, I think I am justified in assuming that, where an early diagnosis is made, the evacuation of the liquor amnii by the hair trocar will be found a safe, easy, and efficient means in lessening or altogether averting the dangers of these hitherto alarming and fatal cases. It may be said that I have somewhat too hastily brought this case before your notice, and that evils may yet arise which might prove that the practice adopted is not so valuable and worthy of imitation as has been assumed. I am most anxious to guard myself against any such imputation, as I am fully aware that nothing is more damaging to the cause of medicine than the publication of so-called successful cases before sufficient time has elapsed to test the efficacy of any plan, especially a new one, resting, as in this instance, upon the case only. Still if any one will well and impartially consider and weigh the whole facts of this case previous to the improvement—aye, more, the speedy and complete disappearance of all the symptoms subsequent to the operation, and the physical changes which have since occurred, I trust I shall be acquitted of anything like precipitancy in the matter, and that this case, at least, will be emphatically declared a complete success.—*Lancet.*

